# Alexithymia and Social Cognition in the General Population: Further Evidence on the Relationship with Theory of Mind, Emotion Recognition, and Empathy

**DOI:** 10.3390/jintelligence14050090

**Published:** 2026-05-21

**Authors:** Aurelia Lo Presti, Marialaura Di Tella, Mauro Adenzato

**Affiliations:** Department of Psychology, University of Turin, 10124 Turin, Italy; aurelia.lopresti@unito.it (A.L.P.); marialaura.ditella@unito.it (M.D.T.)

**Keywords:** alexithymia, social cognition, empathy, Theory of Mind, emotion recognition, ecological validity

## Abstract

Alexithymia has been associated with deficits in social cognition, although findings are inconsistent and often limited by methodological constraints. This study aimed to clarify this relationship using ecologically valid and traditional standardized measures across multiple social-cognitive domains. A total of 163 adults from the general population completed a series of measures, including the Toronto Alexithymia Scale (TAS-20), Questionnaire of Cognitive and Affective Empathy (QCAE), Reading the Mind in the Eyes Test (RMET), Movies for the Assessment of Social Cognition (MASC), and Amsterdam Dynamic Facial Expression Set—Bath Intensity Variations (ADFES-BIV). Results of hierarchical regression analyses revealed that alexithymia facets significantly predicted performance on affective and cognitive empathy (QCAE), and Theory of Mind (MASC total and “No ToM” scores). The only exceptions were affective Theory of Mind (RMET) and recognition of others’ emotions (ADFES-BIV), for which none of the alexithymia facets emerged as significant predictors. The findings suggest that alexithymia is associated with poorer performance in cognitive and affective empathy and contextual Theory of Mind, whereas no significant association emerged for emotion recognition. The results suggest that integrating dynamic and context-rich tasks may be useful for detecting subtle social-cognitive difficulties in individuals with alexithymic traits.

## 1. Introduction

Alexithymia is a psychological construct characterized by difficulties in identifying and describing emotions, and a cognitive style oriented towards concrete and practical aspects of the external environment rather than internal emotional states (Sifneos, 1972; Taylor et al., 1999). Although it plays a role in various psychiatric and medical conditions (Benfante & Romeo, 2023; Di Tella et al., 2018, 2023; Liu et al., 2025; Ozdemir et al., 2025), alexithymia is recognized as existing on a continuum in the non-clinical population (Farina et al., 2021; Mattila et al., 2006; Parker et al., 2008). The authors who first introduced the construct of alexithymia did not explicitly specify whether it should be conceptualized as a dimensional or categorical phenomenon (Nemiah & Sifneos, 1970).

Given that the definition of a construct directly informs its operationalization and measurement, subsequent research sought to clarify the latent structure of alexithymia. In this context, Parker et al. (2008) conducted a taxometric investigation whose results supported conceptualizing alexithymia as a continuous dimension rather than a distinct clinical type. By contrast, authors adopting a categorical perspective have attempted to identify discrete subtypes of alexithymic profiles (Moormann et al., 2008). However, further empirical investigations have provided limited support for such subtype models, failing to identify stable profiles corresponding to the proposed categories (Bagby et al., 2009, 2021). Instead, the profiles emerging from these studies reflected varying degrees of alexithymia severity rather than qualitatively distinct types. Taken together, these findings converge in supporting a dimensional conceptualization of alexithymia as a continuous psychological construct rather than a discrete clinical category (Taylor & Bagby, 2021).

Difficulties in understanding one’s own emotional states have been associated with a diminished capacity for social cognition in both clinical and healthy individuals (Di Tella et al., 2020; Moriguchi et al., 2006). In cognitive psychology, social cognition refers to the ability to represent others’ mental states (their intentions, feelings, and thoughts) and to use these representations to flexibly regulate interpersonal interactions (Adolphs, 2001, 2009). It is commonly conceptualized as comprising at least three core dimensions: the ability to understand the affective and cognitive mental states of others (i.e., Theory of Mind, ToM), the ability to recognize emotional facial expressions, and empathy (Adenzato et al., 2019; Becchio et al., 2006; Decety & Jackson, 2004; Ely & Ambrus, 2025; Enrici et al., 2019; Frith & Frith, 2006). Although the association between alexithymia and social cognition has been studied in the general population, results remain inconsistent regarding the specific domains of social cognition involved (Di Tella et al., 2024; Pisani et al., 2021).

From a theoretical perspective, alexithymia may not affect all components of social cognition equally. Difficulties in identifying and describing one’s own emotional states, along with an externally oriented cognitive style, may be particularly detrimental when social understanding requires integrating contextual cues, inferring others’ mental states, and flexible perspective-taking. In contrast, performance on more specific or less context-dependent tasks may be less consistently associated with alexithymic traits, especially in non-clinical samples where compensatory cognitive resources may support adequate task performance.

Regarding ToM in particular, several studies have demonstrated a negative association between alexithymia and mentalizing abilities (Gosch et al., 2024; Martinez-Sanchez et al., 2017; Sunahara et al., 2022), while others have reported no significant association (Di Tella et al., 2020; Zimmermann et al., 2021). Most results in this area come from studies that use the Reading the Mind in the Eyes Test (RMET; Baron-Cohen et al., 2001) as a measure of ToM (Di Tella et al., 2024; Pisani et al., 2021). In this context, some researchers have recently questioned the psychometric properties of the RMET (Higgins et al., 2026), while others categorize the RMET as a measure of emotion recognition rather than ToM (Murphy & Hall, 2024; Oakley et al., 2016). Specifically, studies by Higgins et al. (2026) and Hafner et al. (2026) attribute some of the limitations of the RMET to the fact that no confirmatory factor analysis study has defined a model that fits well with the structure of the test, a feature with significant implications for both the reliability of statistical measures of internal consistency of the scale in various samples and the interpretation of scores obtained to measure social cognition skills. In contrast, Murphy and Hall (2024) criticized the emphasis placed on the psychometric issue related to the RMET but argue that the RMET could more appropriately be considered a measure of complex emotion recognition rather than ToM. Another aspect relevant to the quality of the RMET as a higher-order ToM measurement tool is that it includes a series of static stimuli, which are not typically recognized as ecological because they are reductive with respect to the complexity of the processes involved in everyday social interactions. Specifically, ecological validity refers to the generalizability of the outcomes beyond the testing context and the representativeness, that is, the degree to which test performance relates to or predicts real-world behaviour (Benito-Ruiz et al., 2022; Dawson & Marcotte, 2017). Despite this, the RMET remains the traditional tool for measuring ToM in both clinical and non-clinical samples. One way to increase the ecological validity of ToM tests is to use stimuli as similar as possible to real-life interactions (Benito-Ruiz et al., 2022). The Movie for the Assessment of Social Cognition (MASC; Dziobek et al., 2006), for example, is a more recent tool that has been suggested to adequately assess ToM in a richer and more dynamic context and is particularly sensitive to subtle social cognitive impairments, due to its distinctive characteristics and good psychometric properties (Dziobek et al., 2006; Tsui et al., 2024).

In recognizing emotional facial expressions, studies using both static (Rosenberg et al., 2020) and dynamic stimuli (Di Tella et al., 2020) have reported less accurate performance in individuals with high alexithymic traits. By contrast, few studies found no significant relationship between alexithymia and the recognition of emotional facial expressions, which Di Tella et al. (2024) attributed to the use of low-ecological-validity tasks or non-standardized experimental measures.

In contrast, results on empathy are more consistent, with several studies showing a negative correlation between alexithymia and empathy (Di Tella et al., 2024).

To address some of the methodological issues outlined above, the present study investigated the relationship between alexithymia and social cognition in the general population using tasks with high ecological validity alongside traditional standardized measures. Beyond this primary objective, the present study also aimed to replicate previously reported findings on the relationship between alexithymia and the three domains of social cognition (Di Tella et al., 2020). Although one objective is to validate previous results, this study differs from the earlier one in several fundamental aspects: the set of social cognition measures employed and the inclusion of the three TAS-20 subscales as independent variables, rather than relying on the total score. Our methodological approach integrates instruments with varying degrees of ecological validity, allowing for a more context-sensitive assessment of social cognition. We hypothesized that alexithymia facets would differentially predict performance on social cognition tasks, thereby providing further insight into how emotional processing difficulties may relate to social cognition. In line with the previous study (Di Tella et al., 2020), we aimed to replicate and expected alexithymia to predict individuals’ empathy scores and lower accuracy in the recognition of facial emotional expressions. Concerning the ToM domain, we hypothesized there would be no association between alexithymia and performance on the RMET. However, given the growing body of literature highlighting the need for more sensitive measures of higher-order ToM, we also included the MASC as a complementary task to examine whether it could provide a more detailed assessment of ToM in our sample and offer additional insight into the predictive role of alexithymia in ToM performance, as observed in previous studies (Gökçen et al., 2016).

## 2. Methods

### 2.1. Participants and Procedure

For the present study, 163 participants were recruited according to the following inclusion criteria: (i) individuals ≥ 18 years of age, (ii) sufficient knowledge of the Italian language, and (iii) no current or previous neurological or psychiatric disorders (assessed with a yes/no question).

All participants took part in the study in the university laboratory. First, they completed a sociodemographic and clinical information form. Subsequently, all measures were presented in random order via online survey software (LimeSurvey, GmbH, Hamburg, Germany, version 6).

The study received approval from the University of Turin Ethics Committee in accordance with the Declaration of Helsinki (protocol no. 0623857), and informed consent was obtained from all participants.

### 2.2. Measures

As part of a larger study, participants completed multiple measures; however, only those instruments relevant to the current study’s aim are described in this section.

**Alexithymia.** The Italian validated version of the *Toronto Alexithymia Scale* (TAS-20; Bagby et al., 1994; Bressi et al., 1996) was used to assess participants’ alexithymia. It is a self-report questionnaire consisting of 20 items divided into three subscales: Difficulty Identifying Feelings (DIF) assesses the individual’s ability to distinguish between specific emotions and bodily sensations associated with physiological arousal; Difficulty Describing Feelings (DDF) evaluates the individual’s capacity to verbalize and express emotions to others; Externally Oriented Thinking (EOT) measures the tendency to focus on concrete, practical, and external aspects of everyday situations rather than on inner emotional states and abstract thinking. Each item is rated on a five-point Likert scale (1 = “strongly disagree,” 5 = “strongly agree”), with higher scores indicating greater alexithymia. For the present study, Cronbach’s alpha was 0.77.

**Empathy.** The Italian version of the *Questionnaire of Cognitive and Affective Empathy* (QCAE; Di Girolamo et al., 2019; Reniers et al., 2011) was used to evaluate empathy. It is a self-report questionnaire comprising 31 items rated on a four-point Likert scale (1 = “strongly agree,” 4 = “strongly disagree”). It evaluates two main dimensions of empathy: cognitive empathy—the ability to understand others’ internal emotional states—and affective empathy, the ability to be sensitive to others’ emotional experiences (Reniers et al., 2011). For the present study, Cronbach’s alpha was 0.73.

**Theory of Mind.** To assess participants’ ability to recognize others’ affective mental states, the RMET (Baron-Cohen et al., 2001) was used. The test consists of 36 photographs depicting eye regions of different human faces. For each item, participants are requested to choose, from four response options, the word that best describes the depicted mental state. In our sample, the Cronbach’s alpha was 0.49.

In conjunction with the RMET, the *Movies for the Assessment of Social Cognition* (MASC; Dziobek et al., 2006; Fossati et al., 2018) was administered as a more ecological instrument to address limitations of the RMET (e.g., Higgins et al., 2026). The MASC is a short film (15 min) that follows four characters as they spend a Saturday evening together. The film is divided into 43 scenes, each of which is associated with a question about the characters’ mental states. Participants are instructed to choose one of four possible response options. One option represents the correct answer to the presented question, while the remaining three provide qualitative indicators of the type of response error associated with participant’s level of ToM: “less ToM” refers to an insufficient ability to recognize others’ mental states; “no ToM” involves the tendency to explain social situations and behaviours by attributing physical causality to them; “excessive ToM” implies the tendency to over-interpret others’ mental states. Each question presented after the clips was accompanied by the photograph of the character it referred to, to reduce the errors in character attribution. In our sample, Cronbach’s alpha was 0.74.

**Facial emotion recognition.** The *Amsterdam Dynamic Facial Expression Set—Bath Intensity Variations* (ADFES-BIV; Wingenbach et al., 2016) was used to measure individuals’ ability to recognize emotional facial expressions. We selected 60 stimuli from the 360 videos of the ADFES-BIV, in which three female and three male actors exhibit nine basic and complex emotions (anger, fear, sadness, contempt, disgust, embarrassment, happiness, pride, and surprise), as well as one neutral expression. The ADFES BIV includes stimuli with three levels of emotional facial expression intensity: low, medium, and high. For the present study, only emotions expressed at medium intensity were selected to ensure an intermediate level of task difficulty. In addition, the stimuli were balanced for actor sex across each specific emotion. The clips were presented in random order without time constraints, and participants were asked to choose the emotion that best matched each facial expression from ten labels. In our sample, Cronbach’s alpha was 0.82.

### 2.3. Statistical Analyses

Statistical analyses were conducted using the Statistical Package for the Social Sciences (SPSS) version 30.0 (IBM SPSS Statistics for Windows, IBM Corp., Armonk, NY, USA).

Normal distribution was assessed using the indices of asymmetry and kurtosis. All variables were within the acceptable range of −2 to +2 (George, 2011). Using G*Power 3.1 (Faul et al., 2009), an *a priori* power analysis was conducted, assuming a medium effect size, power greater than 0.80, and an alpha level of 0.05 for a five-predictor multiple regression analysis. The estimated minimum required sample size was 102 participants. The final sample consisted of 163 participants. Given this final sample size, a sensitivity analysis conducted in G*Power (F tests, linear multiple regression) indicated that, with α = 0.05, power = 0.80, a total sample size of N = 163, and five predictors, the minimum detectable effect size was f^2^ = 0.082. This suggests that the study was sufficiently sensitive to detect small effects.

Descriptive statistics for the total sample were calculated to provide an overview of the respondents’ sociodemographic aspects. Descriptive statistics have been reported as means and standard deviations for continuous variables, and as frequencies with percentages for categorical variables.

To address the main scope of the present study, hierarchical multiple regression analyses were conducted to assess whether alexithymia significantly predicted the different measures of social cognition used. To limit the number of analyses and reduce the risk of Type I error, we focused primarily on global indices for each social-cognitive domain as dependent variables. These included the ADFES-BIV emotion recognition total scores (excluding neutral facial expressions), the RMET total score, the MASC total score, and the QCAE affective and cognitive empathy scores. Additionally, for the MASC, we examined error subtypes (‘Exceed ToM’, ‘Less ToM’, and ‘No ToM’) as they provide theoretically informative qualitative indicators of mentalizing performance. Predictors were entered into the regression model as follows: sociodemographic variables (sex and educational level; first block) and alexithymia facets (second block).

The enter method was used. Effect sizes for regression analyses were calculated and reported as Cohen’s f^2^. Collinearity was assessed using tolerance and Variance Inflation Factor (VIF) statistics.

## 3. Results

### 3.1. Sample Characteristics

Sociodemographic characteristics, as well as data on alexithymia and social cognition measures for the total sample, are presented in Table 1.

### 3.2. Multiple Regressions

To examine whether alexithymic facets were significant predictors of social cognition measures, six hierarchical multiple regression analyses were conducted. The ADFES-BIV emotion recognition total score, RMET, MASC scores, and QCAE affective and cognitive empathy scores were entered as dependent variables in the regression analyses.

For empathy, the full model including sex, educational level, and alexithymia facets predicting affective empathy (Model 2) was statistically significant, adjusted R^2^ = 0.150 (SE = 4.812), F(5, 157) = 6.717, *p* < .001. This corresponds to a medium effect size (f^2^ = 0.18). In this model, the DIF (β = 0.438, *p* < .001) and DDF (β = −0.225, *p* = .007) subscales of the TAS-20 were significant predictors of QCAE “Affective” scores (Table 2). Similarly, the full model including sex, educational level, and alexithymia facets predicting cognitive empathy was statistically significant, adjusted R^2^ = 0.205 (SE = 6.506), F(5, 157) = 9.340, *p* < .001, indicating a moderate effect size (f^2^ = 0.26). Here, the DDF (β = −0.265, *p* = .001) and EOT (β = −0.325, *p* < .001) subscales were significant predictors of QCAE “Cognitive” scores (Table 2).

Regarding ToM abilities, the full model including sex, educational level, and alexithymia facets predicting the RMET total score (Model 2) was not statistically significant, adjusted R^2^ = −0.005 (Standard Error, SE = 3.524), F(5, 157) = 0.832, *p* = .529, indicating a negligible effect size. No significant predictive effects on RMET scores were observed for any variables in the model (Table 3).

Conversely, the full model, including sex, educational level, and alexithymia facets predicting MASC total score (Model 2), was statistically significant, adjusted R^2^ = 0.056 (SE = 5.391), F(5, 157) = 2.904, *p* = .015, corresponding to a small effect size (f^2^ = 0.06). The only significant predictor was EOT (β = −0.222, *p* = .007) (Table 3). Similarly, the full model including sex, educational level, and alexithymia facets predicting the MASC “Exceed ToM” score (Model 2) was statistically significant, adjusted R^2^ = 0.041 (Standard Error, SE = 3.014), F(5, 157) = 2.394, *p* = .040, with a small effect size (f^2^ = 0.04). Sex (β = 0.203, *p* = .011) was the only significant predictor of MASC “Exceed ToM” scores (Supplementary Materials Table S1). However, the full model, including sex, educational level, and alexithymia facets predicting the MASC “Less ToM” score (Model 2), was not statistically significant, adjusted R^2^ = −0.003 (Standard Error, SE = 3.401), F(5, 157) = 0.914, *p* = .474, indicating a negligible effect size. None of the variables in the model significantly predicted MASC “Less ToM” scores (Supplementary Materials Table S2). For the last MASC score, the full model including sex, educational level, and alexithymia facets predicting the MASC “No ToM” score (Model 2) was statistically significant, adjusted R^2^ = 0.058 (Standard Error, SE = 2.120), F(5, 157) = 3.003, *p* = .013, corresponding to a small effect size (f^2^ = 0.06). The only significant predictor of the MASC “No ToM” score was the EOT subscale score of the TAS-20 (β = 0.267, *p* = .001) (Supplementary Materials Table S3).

Finally, regarding emotion recognition, the full model including sex, educational level, and alexithymia facets predicting the ADFES-BIV emotion recognition total score (Model 2) was not statistically significant, adjusted R^2^ = 0.014 (Standard Error, SE = 6.803), F(5, 157) = 1.7, *p* = .204, indicating a very small effect size (f^2^ = 0.01). None of the variables in the model significantly predicted ADFES-BIV emotion recognition total scores (Table 4).

In all regression analyses, the tolerance and VIF statistics indicated no confounding interactions among the variables.

Collectively, the hierarchical regressions showed that alexithymia significantly predicted empathy and context-rich ToM performance. In contrast, no significant association was found for affective ToM (RMET) or for the recognition of others’ emotions.

## 4. Discussion

This study aimed to clarify the association between alexithymia and social cognition in the general population, addressing certain methodological limitations of previous research.

Consistent with our initial hypothesis, the results showed that facets of alexithymia were differently related to participants’ performance in the three core dimensions of social cognition. Specifically, alexithymia dimensions significantly predicted ToM, and cognitive and affective empathy scores, but not facial emotion recognition accuracy.

Regarding ToM, alexithymic traits negatively predicted individuals’ performance on ToM as measured by the MASC, but not by the RMET, for which no significant effect of alexithymia was found. These results are consistent with our expectations and replicate the findings of Di Tella et al. (2020). The reliability indices of the RMET in our sample were unexpectedly low, limiting the interpretability of the findings. Although the RMET remains the traditional tool for measuring ToM, mounting empirical evidence has raised concerns about its psychometric robustness. To date, confirmatory factor-analytic investigations have failed to identify a model that demonstrates adequate fit to the proposed instrument structure (Higgins et al., 2026; Hafner et al., 2026). As highlighted by Higgins et al. (2026), inter-item correlations are generally weak, suggesting that responses to individual RMET items poorly predict responses to other items within the scale. This pattern raises questions about whether the instrument captures a coherent, specific socio-cognitive ability that accounts for overall test performance. Such findings undermine the internal consistency and, more broadly, the internal validity of the measure. Indeed, studies employing the RMET across different populations have reported internal consistency indices (e.g., Cronbach’s α, McDonald’s ω) that are marginally acceptable or fall below commonly recommended thresholds (Higgins et al., 2026). Furthermore, evidence of a non-unidimensional factor structure challenges the appropriateness of reliability estimates grounded in the assumption of unidimensionality (e.g., Cronbach’s α, McDonald’s ω; Hafner et al., 2026), thereby complicating the interpretation of scores. In the present sample, Cronbach’s α was 0.49, a value consistent with previously documented psychometric limitations of the instrument. Although this result aligns with the broader literature, it nonetheless requires caution in interpreting the findings derived from the RMET scores. Beyond the psychometric limitations of the instrument, the inconsistency of findings observed in this and other studies, as highlighted in recent systematic reviews examining the relationship between alexithymia and ToM using the RMET (Di Tella et al., 2024; Pisani et al., 2021), may reflect differences in the underlying cognitive processes required by the two tasks. Specifically, performance on the RMET appears to be particularly influenced by other cognitive factors, including intelligence and verbal intelligence (Baker et al., 2014; Hafner et al., 2026; Higgins et al., 2026). Moreover, several authors have argued that the RMET primarily captures low-context emotion recognition rather than higher-order mentalizing (Murphy & Hall, 2024; Oakley et al., 2016; Pisani et al., 2021). As Pisani et al. (2021) highlighted, the association between alexithymia and ToM, as measured by the RMET, may be partly influenced by the task’s emotion recognition component. They suggest that distinct cognitive strategies may be employed during RMET performance, and that the degree to which emotion recognition abilities affect task outcomes likely varies across populations. This variability may help explain the inconsistent findings reported in the literature. This interpretation is also consistent with our findings on the recognition of emotional facial expressions measured with the ADFES-BIV, for which alexithymia did not significantly predict performance, as well as with the results observed for the RMET. The MASC, by contrast, offers a more dynamic and context-rich evaluation of ToM and appears more sensitive to subtle social-cognitive deficits (Dziobek et al., 2006; Tsui et al., 2024). It requires participants to distinguish between characters’ thoughts and beliefs and their emotional states, integrating multiple perspectives with contextual cues and drawing on the verbal content of conversations as well as prosodic and nonverbal information. In this sense, as noted above, the specific features of the MASC (i.e., the use of dynamic rather than static stimuli, the depiction of interactions among multiple characters, and the representation of everyday life situations) confer greater ecological validity compared to the RMET. Our results strengthen the argument that alexithymia may be associated with difficulties in the cognitive processes underlying social understanding, particularly when tasks require the integration of contextual information and flexible perspective-taking. In particular, among the TAS-20 subscales, EOT emerged as a factor that negatively predicted MASC scores. EOT refers to a general tendency to focus on concrete aspects of the external world rather than on emotional states and internal thoughts. In examining the mechanisms through which alexithymia moderates the cognitive processing of emotional information, Luminet et al. (2024) conducted an integrative review in which they categorize the included studies into five domains (i.e., memory, language, attention, appraisal, and behaviour) and examined the impact of different facets of alexithymia (e.g., DIF, DDF, and EOT). The authors aimed to determine whether the dysfunctions observed in individuals with alexithymic traits reflected specific processing deficits or a more general tendency to over-respond to emotional contexts—namely, heightened emotional reactivity despite difficulties in identifying and describing one’s feelings—reflecting a mismatch between internal affective experience and outward emotional expression with behavioural consequences (e.g., avoidance, suppression, or dysregulated reactions). Overall, EOT was found to be mainly associated with specific deficits in the cognitive processing of emotional information across multiple domains (i.e., memory and language), leading to deficits in the interpretation and elaboration of emotional information. The authors suggested that difficulties in verbally elaborating feelings, combined with an externally oriented cognitive style, hinder the integration of emotions with thoughts, memories, and goals. This, in turn, may contribute to reduced awareness of one’s own emotional state (Luminet et al., 2024). In the specific context of ToM, difficulties in processing one’s own emotional experiences may be a plausible mechanism underlying the association between alexithymia and lower performance on ToM tasks. Notably, the capacity to reflect on one’s own mental states is closely linked to the ability to interpret the mental states of others (Decety & Sommerville, 2003). Reduced engagement in internal self-monitoring processes may, in turn, be associated with difficulties in attributing and reflecting on the mental states of others.

Contrary to our initial hypothesis, no effect of alexithymia on facial emotion recognition was found. As noted above, previous studies investigating the specific relationship between alexithymia and the recognition of emotional facial expressions have reported conflicting results. More recently, Willis et al. (2025) suggested that the type of stimulus moderates this association: while static emotional stimuli are more difficult for alexithymic individuals to categorize, their performance is comparable to that of non-alexithymic individuals when presented with dynamic stimuli. In line with this interpretation, the results of the present study show that alexithymia did not predict participants’ overall performance in the recognition of emotional facial expressions when they were confronted with the dynamic stimuli of the ADFES-BIV. However, it should be noted that dynamic stimuli depicting basic emotions were also used in the previous study by Di Tella et al. (2020). Therefore, the discrepancy between the findings may be attributable to differences in sample characteristics.

Finally, the data from the current study replicate the well-documented negative relationship between empathy and alexithymia. Studies investigating the relationship between alexithymia and empathy generally agree in reporting a negative association between alexithymic traits and overall empathy. However, inconsistencies emerge when distinguishing between cognitive and affective empathy: some studies report effects only for cognitive empathy, others only for affective empathy, and still others for both (Di Tella et al., 2024; Pisani et al., 2021). Affective empathy refers to the ability to *vicariously experience* the emotional states of others. Meanwhile, cognitive empathy is defined as the capacity to *understand* others’ emotional experiences and feelings (Reniers et al., 2011) and is closely related to the specific ToM difficulties described above. From a neuroscientific point of view, the mechanisms involved in empathic processes allow us to distinguish between a basic emotional contagion system (affective empathy) and a more advanced cognitive perspective-taking system (cognitive empathy; Shamay-Tsoory et al., 2009). The present study found that alexithymic facets are associated with lower scores on both cognitive and affective empathy scales. More specifically, the DDF and EOT subscales predicted lower levels of cognitive empathy, while the DDF subscale predicted lower levels of affective empathy. Counterintuitively, the DIF subscale positively predicted affective empathy. This finding suggests that difficulty identifying one’s own emotions does not necessarily imply reduced affective responsiveness to others and may instead coexist with heightened self-reported affective resonance. One possible interpretation is that this pattern reflects emotional contagion or undifferentiated affective arousal rather than more regulated empathic responding. Therefore, this association should be interpreted with caution and requires replication in future studies.

This study has some limitations. First, its cross-sectional design precludes establishing causal relationships among the variables; these should be investigated in future longitudinal studies. Second, empathy and alexithymia were assessed using self-report measures in this sample. Nevertheless, the instruments used demonstrate high validity and effectiveness in assessing the constructs they are intended to measure (Di Girolamo et al., 2019; Taylor et al., 2003). Third, we did not administer any structured clinical interview or standardized self-report measure to assess participants’ current or past psychopathology. Instead, clinical history was assessed through a single dichotomous (yes/no) self-report question. Fourth, because the final sample exceeded the *a priori* minimum, the study may have been sensitive enough to detect very small effects. Therefore, statistically significant findings with small effect sizes should be interpreted with caution and ideally cross-validated in an independent sample. Finally, the generalizability of our findings is limited by the fact that the study was conducted in a university laboratory setting and that all participants were native Italian speakers. Although the sample was not composed exclusively of university students, the recruitment context may nonetheless restrict the extent to which the results can be generalized to more diverse populations.

Despite these limitations, the present study suggests that higher levels of alexithymia are associated with poorer performance in higher-order social-cognitive functions, particularly cognitive and affective empathy and contextual ToM. These findings underline the importance of incorporating dynamic, context-rich assessments in both research and clinical assessment. From a translational perspective, interventions fostering emotional awareness and perspective-taking may represent promising avenues for improving interpersonal functioning in individuals with elevated alexithymic traits. Future longitudinal and neurocognitive studies are needed to clarify causal pathways and to develop targeted social-cognitive training protocols.

## Figures and Tables

**Table 1:** Descriptive statistics of the sociodemographic and social cognition variables (N = 163).

	Mean (SD)	N (%)	Range
**Sex (F)**		93 (57.1)	
**Age**	44.88 (19.07)		19–84
**Occupation**			
Student	36	22.1	
Employed	81	49.7	
Not employed	14	8.6	
Retired	32	19.6	
**Marital status**			
Single	74	45.4	
Cohabitant	14	8.6	
Married	50	30.7	
Separated/Divorced	15	9.2	
Widowed	10	6.1	
**Educational level**	14.95 (3.37)		8–22
Middle school	14	8.6	
High school	67	41.1	
Bachelor	31	19	
Master’s degree	41	25.2	
Post-graduated	10	6.1	
**RMET**	26.20 (3.51)		15–33
**MASC Total Score**	29.90 (5.55)		10–40
**MASC Exceed ToM**	6.94 (3.08)		1–17
**MASC Less ToM**	5.28 (3.4)		0–17
**MASC No ToM**	2.98 (2.18)		0–11
**QCAE Affective**	34.23 (5.22)		22–47
**QCAE Cognitive**	59.99 (7.29)		41–75
**TAS-20 Total core**	43.58 (10.32)		25–73
**TAS-20 DIF**	15.45 (5.53)		7–32
**TAS-20 DDF**	12.75 (4.43)		5–24
**TAS-20 EOT**	15.37 (4.41)		8–26

F = Female; SD = Standard deviation; RMET = Reading the Mind in the Eyes Test; MASC = Movie for the Assessment of Social Cognition; QCAE = Questionnaire of Cognitive and Affective Empathy; TAS-20 = Twenty-item Toronto Alexithymia Scale; DIF = Difficulty Identifying Feelings subscale of the Toronto Alexithymia Scale; DDF = Difficulty Describing Feelings of the Toronto Alexithymia Scale; EOT = Externally Oriented Feelings subscale of the Toronto Alexithymia Scale.

**Table 2:** Hierarchical multiple regressions predicting QCAE Affective and Cognitive scores from sociodemographic variables and alexithymia facets (N = 163).

*Empathy*								
Predictor Variables	B	β	t	95% CI	Adj R^2^	F	ΔR^2^	ΔF
**QCAE Affective**								
***Model 1***					0.005	1.398	0.017	1.398
**Sex**	−1.253	−0.119	−1.513	−2.888; 0.383				
**Educational level**	−0.068	−0.044	−0.555	−0.308; 0.173				
***Model 2***					0.150	6.717 **	0.159	10.103 **
**Sex**	−0.589	−0.056	−0.752	−2.138; 0.960				
**Educational level**	−0.071	−0.046	−0.604	−0.305; 0.162				
**TAS_DIF**	0.413	0.438	5.325 **	0.260; 0.566				
**TAS_DDF**	−0.266	−0.225	−2.714 **	−0.459; 0.072				
**TAS_EOT**	−0.132	−0.111	−1.442	−0.312; 0.049				
**QCAE Cognitive**								
***Model 1***					0.007	1.588	0.019	1.588
**Sex**	−1.701	−0.116	−1.471	−3.984; 0.582				
**Educational level**	0.196	0.090	1.149	−0.140; 0.532				
***Model 2***					0.205	9.340 **	0.210	14.245 **
**Sex**	−0.755	−0.051	−0.712	−2.850; 1.339				
**Educational level**	−0.115	−0.053	−0.720	−0.431; 0.201				
**TAS_DIF**	−0.071	−0.054	−0.678	−0.278; 0.136				
**TAS_DDF**	−0.437	−0.265	−3.299 **	−0.698; −0.175				
**TAS_EOT**	−0.538	−0.325	−4.359 **	−0.782; −0.294				

CI = Confidence Interval; QCAE = Questionnaire of Cognitive and Affective Empathy; TAS-20 = Twenty-item Toronto Alexithymia Scale; DIF = Difficulty Identifying Feelings subscale of the Toronto Alexithymia Scale; DDF = Difficulty Describing Feelings of the Toronto Alexithymia Scale; EOT = Externally Oriented Feelings subscale of the Toronto Alexithymia Scale. ** *p* < .01.

**Table 3:** Hierarchical multiple regressions predicting RMET and MASC total scores from sociodemographic variables and alexithymia facets (N = 163).

*Theory of Mind*								
Predictor Variables	B	β	t	95% CI	Adj R^2^	F	ΔR^2^	ΔF
**RMET**								
***Model 1***					−0.001	0.917	0.011	0.917
**Sex**	−0.483	−0.068	−0.863	−1.588; 0.622				
**Educational level**	0.093	0.089	1.125	−0.070; 0.255				
***Model 2***					−0.005	0.832	0.014	0.777
**Sex**	−0.448	−0.063	−0.780	−1.582; 0.687				
**Educational level**	0.059	0.056	0.679	−0.112; 0.230				
**TAS_DIF**	−0.062	−0.098	−1.091	−0.174; 0.050				
**TAS_DDF**	0.006	0.007	0.080	−0.136; 0.147				
**TAS_EOT**	−0.056	−0.071	−0.843	−0.189; 0.076				
**MASC Total score**								
***Model 1***					0.027	3.272 *	0.039	3.272 *
**Sex**	−0.869	−0.078	−0.999	−2.588; 0.849				
**Educational level**	0.313	0.190	2.444	0.060; 0.566				
***Model 2***					0.056	2.904 *	0.045	2.593
**Sex**	−0.461	−0.041	−0.524	−2.196; 1.275				
**Educational level**	0.219	0.133	1.655	−0.042; 0.481				
**TAS_DIF**	−0.037	−0.037	−0.427	−0.209; 0.134				
**TAS_DDF**	0.043	0.034	0.390	−0.174; 0.259				
**TAS_EOT**	−0.279	−0.222	−2.726 **	−0.481; −0.077				

CI = Confidence Interval; RMET = Reading the Mind in the Eyes Test; MASC = Movie for the Assessment of Social Cognition; TAS-20 = Twenty-item Toronto Alexithymia Scale; DIF = Difficulty Identifying Feelings subscale of the Toronto Alexithymia Scale; DDF = Difficulty Describing Feelings of the Toronto Alexithymia Scale; EOT = Externally Oriented Feelings subscale of the Toronto Alexithymia Scale. * *p* < .05. ** *p* < .01.

**Table 4:** Hierarchical multiple regressions predicting ADFES-BIV emotion recognition total score from sociodemographic variables and alexithymia facets (N = 163).

*Emotion Recognition*—ADFES-BIV								
Predictor Variables	B	β	t	95% CI	Adj R^2^	F	ΔR^2^	ΔF
***Model 1***					0.025	3.061	0.037	3.061
**Sex**	−1.932	−0.140	−1.795	−4.057; 0.193				
**Educational level**	0.297	0.146	1.876	−0.016; 0.610				
***Model 2***					0.014	1.467	0.008	0.426
**Sex**	−1.783	−0.129	−1.608	−3.973; 0.407				
**Educational level**	0.251	0.124	1.501	−0.079; 0.581				
**TAS_DIF**	0.020	0.016	0.181	−0.197; 0.236				
**TAS_DDF**	−0.128	−0.083	−0.926	−0.402; 0.145				
**TAS_EOT**	−0.057	−0.037	−0.440	−0.312; 0.198				

CI = Confidence Interval; ADFES-BIV = Amsterdam Dynamics Facial expression Set—Bath Intensity Variations; TAS-20 = Twenty-item Toronto Alexithymia Scale; DIF = Difficulty Identifying Feelings subscale of the Toronto Alexithymia Scale; DDF = Difficulty Describing Feelings of the Toronto Alexithymia Scale; EOT = Externally Oriented Feelings subscale of the Toronto Alexithymia Scale.

## Data Availability

The data are available on request from the corresponding author.

## Supplementary Materials

The following supporting information can be downloaded at https://www.mdpi.com/article/10.3390/jintelligence14050090/s1.
