# Supplementary material for: Alexithymia and Social Cognition in the General Population: Further Evidence on the Relationship with Theory of Mind, Emotion Recognition, and Empathy
Source: J Intell. 2026 May 21;14(5):90. doi: 10.3390/jintelligence14050090 (PMC13208129; doi:10.3390/jintelligence14050090)
Supplement: Supplementary file 1 [file jintelligence-14-00090-s001.zip › jintelligence-4128817-supplementary.pdf]

Table S1. Hierarchical multiple regressions predicting MASC Exceed Theory of Mind scores from sociodemographic variables and alexithymia facets (N = 163).

MASC EXCEED TOM

Predictor variables	B	β	t	95% CI	Adj R^2	F	ΔR^2	ΔF
Model 1					0,047	4,983**	0,059	4,983**
**Sex**	1,234	0,199	2,582*	0,290; 2,178				
**Educational level**	-0,146	-0,159	-2,069*	-0,285; -0,007				
Model 2					0,041	2,394*	0,012	0,687
**Sex**	1,259	0,203	2,562*	0,289; 2,229				
**Educational level**	-0,135	-0,148	-1,826	-0,282; 0,011				
**TAS_DIF**	0,061	0,109	1,248	-0,035; 0,157				
**TAS_DDF**	-0,062	-0,089	-1,008	-0,183; 0,059				
**TAS_EOT**	0,029	0,042	0,514	-0,084; 0,142				

CI = Confidence Interval; TOM = Theory of Mind; MASC EXCEED TOM = Exceed ToM scores of the Movie for the Assessment of Social Cognition; TAS-20 = Twenty-item Toronto Alexithymia Scale; DIF = Difficulty Identifying Feelings subscale of the Toronto Alexithymia Scale; DDF = Difficulty Describing Feelings of the Toronto Alexithymia Scale; EOT = Externally Oriented Feelings subscale of the Toronto Alexithymia Scale.

Table S2. Hierarchical multiple regressions predicting MASC Less Theory of Mind scores from sociodemographic variables and alexithymia facets (N = 163).

MASC LESS TOM

Predictor variables	B	β	t	95% CI	Adj R^2	F	ΔR^2	ΔF
Model 1					-0,008	0,367	0,005	0,367
**Sex**	-0,125	-0,018	-0,231	-1,197; 0,946				
**Educational level**	-0,064	-0,063	-0,797	-0,221; 0,094				
Model 2					-0,003	0,914	0,024	1,277
**Sex**	-0,361	-0,053	-0,651	-1,456; 0,734				
**Educational level**	-0,033	-0,032	-0,390	-0,198; 0,132				
**TAS_DIF**	-0,048	-0,078	-0,875	-0,156; 0,060				
**TAS_DDF**	0,018	0,023	0,259	-0,119; 0,155				
**TAS_EOT**	0,115	0,149	1,777	-0,013; 0,242				

CI = Confidence Interval; TOM = Theory of Mind; MASC LESS TOM = Less ToM scores of the Movie for the Assessment of Social Cognition; TAS-20 = Twenty-item Toronto Alexithymia Scale; DIF = Difficulty Identifying Feelings subscale of the Toronto Alexithymia Scale; DDF = Difficulty Describing Feelings of the Toronto Alexithymia Scale; EOT = Externally Oriented Feelings subscale of the Toronto Alexithymia Scale.

Table S3. Hierarchical multiple regressions predicting MASC No ToM scores from sociodemographic variables and alexithymia facets (N = 163).

MASC NO TOM								
**Predictor variables**	**B**	**β**	**t**	**95% CI**	**Adj R^2**	**F**	**ΔR^2**	**ΔF**
Model 1					0,006	1,523	0,019	1,523
**Sex**	-0,007	-0,002	-0,021	-0,691; 0,677				
**Educational level**	-0,088	-0,137	-1,734	-0,189; 0,012				
Model 2					0,058	3,003*	0,069	3,934**
**Sex**	-0,207	-0,047	-0,601	-0,890; 0,475				
**Educational level**	-0,040	-0,061	-0,759	-0,142; 0,063				
**TAS_DIF**	0,017	0,043	0,493	-0,051; 0,084				
**TAS_DDF**	-0,002	-0,004	-0,050	-0,087; 0,083				
**TAS_EOT**	0,132	0,267	3,292**	0,053; 0,212				

CI = Confidence Interval; TOM = Theory of Mind; MASC NO TOM = No ToM scores of the Movie for the Assessment of Social Cognition; TAS-20 = Twenty-item Toronto Alexithymia Scale; DIF = Difficulty Identifying Feelings subscale of the Toronto Alexithymia Scale; DDF = Difficulty Describing Feelings of the Toronto Alexithymia Scale; EOT = Externally Oriented Feelings subscale of the Toronto Alexithymia Scale.
